# Strong Dimensional and Structural Dependencies of Hot Carrier Effects in InGaAs Nanowires: Implications for Photovoltaic Solar Cells

**DOI:** 10.1021/acsanm.3c05041

**Published:** 2024-01-23

**Authors:** Hamidreza Esmaielpour, Nabi Isaev, Imam Makhfudz, Markus Döblinger, Jonathan J. Finley, Gregor Koblmüller

**Affiliations:** † Walter Schottky Institut, TUM School of Natural Sciences, Technical University of Munich, 85748 Garching, Germany; ‡ IM2NP, UMR CNRS 7334, Aix-Marseille Université, Marseille 13013, France; § Department of Chemistry, Ludwig-Maximilians-University Munich, Munich 81377, Germany

**Keywords:** nanowires, hot carrier effects, thermalization mechanism, Auger recombination, selective area epitaxy

## Abstract

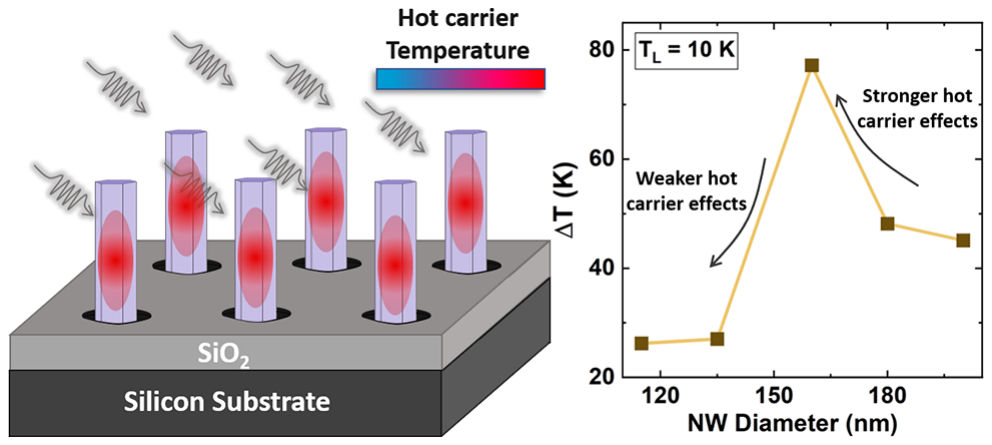

III–V nanowire structures are among the promising material systems with applications in hot carrier solar cells. These nanostructures can meet the requirements for such photovoltaic devices, i.e., the suppression of thermalization loss, an efficient hot carrier transport, and enhanced photoabsorption thanks to their unique one-dimensional (1D) geometry and density-of-states. Here, we investigate the effects of spatial confinement of photogenerated hot carriers in InGaAs-InAlAs core–shell nanowires, which presents an ideal class of hot carrier solar cell materials due to its suitable electronic properties. Using steady-state photoluminescence spectroscopy, our study reveals that by increasing the degree of spatial confinement and Auger recombination, the effects of hot carriers increase, which is in good agreement with theoretical modeling. However, for thin nanowires, the temperature of hot carriers decreases as the effects of crystal disorder increase. This observation is confirmed by probing the extent of the disorder-induced Urbach tail and linked to the presence of a higher density of stacking defects in the limit of thin nanowires. These findings expand our knowledge of hot carrier thermalization in nanowires, which can be applied for designing efficient 1D hot carrier absorbers for advanced-concept photovoltaic solar cells.

## Introduction

Relaxation of hot carriers after photogeneration is responsible for a large fraction of energy loss in photovoltaic (PV) solar cells, but yet it is one of the least understood phenomena in semiconductors. According to the detailed balance analysis, the maximum power conversion efficiency of single-junction solar cells under one sun condition is about 33%, known as the Shockley–Queisser limit.^1^ Recent developments in PV solar cells have made it possible to achieve efficiencies very close to this upper limit.^2^ To further boost the efficiency of this technology beyond the upper theoretical limit, it is inevitable to eliminate some major loss mechanisms, such as thermalization of hot carriers, in PV devices.

Suppressing the thermalization of hot carriers and increasing the efficiency of solar cells by converting the energy loss due to thermalization to electricity are the main objectives of hot carrier solar cells (HCSCs), which aim for 67% efficiency under one sun condition.^3^ As proposed by Ross and Nozik,^4^ this approach is realized by (1) designing efficient hot carrier absorbers that can inhibit the thermalization mechanism and (2) fast extraction of hot carriers via energy-selective contacts at energy levels larger than the bandgap.

In polar semiconductors, interactions of electrons with longitudinal optical (LO) phonons, known as Fröhlich interactions, are one of the main relaxation pathways for hot electrons.^5^ A promising method for reducing the relaxation rates of hot populations is the spatial confinement of charged particles, which can make phonon emission and absorption rates to be comparable, leading to the phonon bottleneck effect.^6^ Under such conditions, a nonequilibrium distribution of phonons, called hot phonons, arises, which can lower the relaxation rates of hot carriers in semiconductors.^7−9^

Nanowires (NWs) are one type of nanostructures that can meet both requirements for HCSC designs.^10^ First, their 1D geometries can enable greater spatial confinement of hot carriers compared to bulk and two-dimensional (2D) quantum well structures.^11^ Second, their narrow and discrete density-of-states can reduce hot carrier thermalization rates at the contacts without the need to design double barrier structures, as is the case with quantum well and quantum dot designs for hot carrier extraction.^12,13^ Furthermore, the transport of hot carriers along the NWs in axial heterostructures exhibits superior performance because carriers can only move along the 1D configuration of the NWs.^14^ Another advantage of NWs assembled in array-geometries is their applications in light management, which can increase photoabsorption of solar cell absorbers beyond the ray optics limit.^15,16^ This application is significant for HCSCs, as the diffusion length of hot carriers is small (only a few hundred nanometers^17^) and it is important to design thin films with high photoabsorptivity. In addition to these favorable properties of NWs for HCSC applications, these nanostructures have been applied in various optoelectronic and electronic devices, including photodetectors,^18^ lasers,^19^ as well as high-mobility transistor,^20,21^ and thermoelectric devices.^22^

One of the first demonstrations of hot carrier extraction in NWs was reported by Limpert et al.^23^ using InAs/InP heterostructure NWs, in which photogenerated hot carriers in the InAs absorber were extracted from the InP barrier by thermionic emission. Evidence of hot carrier extraction in this design was observed by an open-circuit voltage greater than the bandgap energy (divided by the Coulomb charge) of the InAs absorber.

Hot carrier effects in NWs have been further studied using steady-state and time-resolved optical spectroscopy.^24−28^ Tedeschi et al.^24^ studied the effects of hot carriers in NWs under continuous wave excitation and found that there is a reciprocal relationship between hot carrier temperature and NW diameter. One of the main characteristics of NWs is their large surface-to-volume ratio, which can improve photoabsorption for solar cell applications.^29^ However, the large surface-to-volume ratio of NWs can increase the influence of surface states on hot carriers and their thermalization rates.^30^ Sandner et al.^25^ studied therefore the effects of surface passivation of NWs on their hot carrier properties using transient absorption pump–probe spectroscopy. Comparison of bare-core InAs and InAs-AlAsSb core–shell NWs showed that the passivation layer reduces Fermi-level pinning effects (lowering band bending) at the interface of the InAs absorber, while the rate of Auger recombination increases, both of which lead to an increase in hot carrier effects. While such studies are limited to InAs NWs and their peculiar surface properties, a more generalistic investigation of hot carrier effects in core–shell NWs is needed to reveal important influences by size dimension and other material-inherent properties, such as microstructure and defects.

In this work, a comprehensive study of the impact of dimensional and microstructural properties on hot carrier effects is demonstrated based on the technologically relevant InGaAs/InAlAs core–shell NW system. InGaAs NWs are among the most appealing candidate materials due to their tunable bandgap, especially toward 0.7 eV, which is the optimum bandgap energy of HCSCs with a relatively small thermalization coefficient (50 W/(K·cm^2^)),^31^ high carrier mobility,^32^ and high absorption coefficient.^16^ Recently, it has been shown that InGaAs/InAlAs heterostructured designs have promising applications in valley-photovoltaic solar cells.^33,34^ However, the NW design of such compounds exhibit polytypism (intermixing of zinc-blende and wurtzite crystals) commonly observed in most III–V semiconductor NWs.^35,36^ Our studies are enabled by realizing high-uniformity arrays of selective-area grown (SAG) InGaAs NWs passivated by thin epitaxial InAlAs shells, where wide tunability in size dimension (NW diameter) is achieved by accurately tuning the geometry of the SAG pattern. The hot carrier properties of the NW arrays are assessed by photoluminescence (PL) spectroscopy, and the temperature of hot carriers is determined by analyzing the PL spectra using the generalized Planck’s radiation law while taking the diameter-dependent photoabsorption of individual arrays into account. To understand the origin and dynamics of the observed hot carrier effects, detailed discussions are provided in the framework of theoretical analysis of hot carrier thermalization rates in relation to NW diameter, recombination mechanisms, and microstructural properties of the epitaxially grown NWs.

## Results and Discussion

The NW-arrays are grown by solid-source molecular beam epitaxy (MBE) using a catalyst-free (vapor–solid) SAG approach on prepatterned SiO_2_/Si(111) substrate. Hereby, periodic arrays of lithographically defined mask openings (fabricated by electron beam lithography) allow NWs to only form in the openings with high selectivity, where the size and arrangement of the NWs is controlled by the mask opening size.^37^ We tune the mask opening size from 50 to 180 nm at a fixed pitch of 0.5 μm and grow NW-arrays at fixed composition, i.e., In_0.2_Ga_0.8_As/In_0.2_Al_0.8_As, by adopting previously established growth parameters.^38,39^ In this way, a large number of NW fields with different dimensions can be obtained within a single growth run.

**Figure 1:**
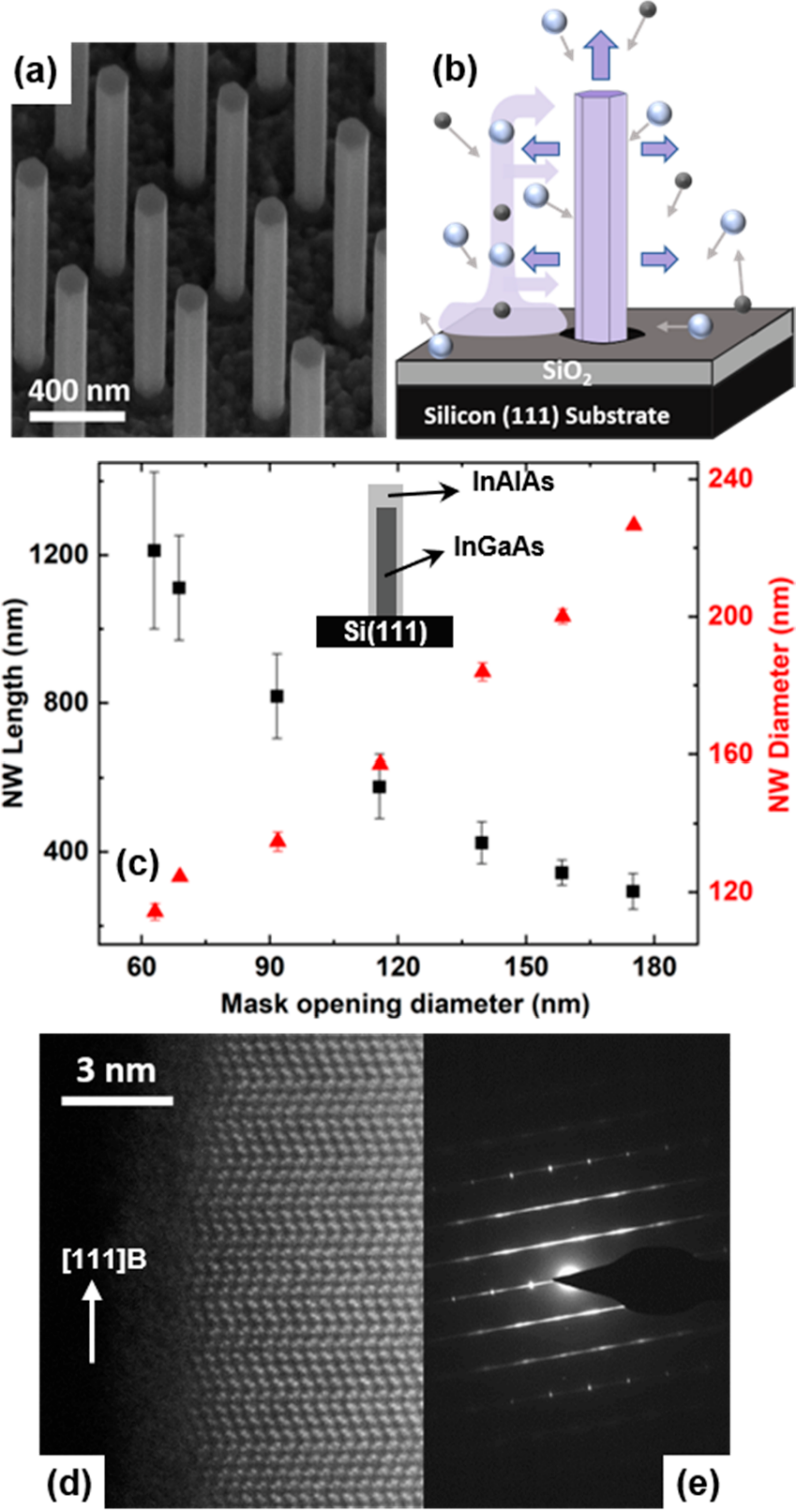
(a) SEM image in bird-eye view (45°) of a representative InGaAs/InAlAs NW array. (b) Schematic of NW growth via the catalyst-free selective-area growth method. (c) Dependence of the length (black) and diameter (red) of the NWs versus the mask opening diameter of the prepatterned SiO_2_/Si(111) substrate. (d) TEM image and (e) corresponding SAD pattern of a typical NW, showing a microstructure with ultrashort ZB rotational twin domains and a high density of stacking defects.

The growth of the InGaAs core is performed for 60 min at a temperature of 590 °C and a V/III ratio of 88, resulting in an indium (In) content of 20% as verified by high-resolution X-ray diffraction (HR-XRD); see Figure S1 in the Supporting Information. The NWs are passivated by a thin layer (10 nm) of InAlAs, grown at 570 °C and V/III ratio of 150. This gives us an almost identical In content of ∼20% in the cladding layer, as shown in the energy dispersive X-ray (EDX) map in Figure S2 in the Supporting Information, facilitating close lattice matching between the core and shell through fully coherent growth. As noted in ref (39), the InAlAs passivation helps to enhance the luminescence efficiency of the InGaAs NW core. Figure 1a shows a representative scanning electron microscopy (SEM) image of an as-grown NW-array, as obtained, for example, from a field with a mask opening diameter of 90 nm. The image demonstrates the excellent selective area growth with high growth yield (∼80%) and minimal variation in NW size dimensions across the array. A schematic of the NW growth through the catalyst-free selective-area growth process is shown in Figure 1b. Here, the growth proceeds predominantly in the axial direction via incorporation of diffusing species to the NW tip but also radially via incorporation on the NW sidewall surfaces (group III and group V species are indicated by blue and gray symbols, respectively, as shown in Figure 1b).^40^

Figure 1c shows the length and diameter dependence of the core–shell NWs as a function of the mask opening diameter. It is seen that by increasing the size of the mask opening, the length of the NWs decreases while their diameter increases. Note that this trend purely reflects the dynamics of the InGaAs core growth, as previously observed^38^ and as confirmed by reference NW-arrays without passivation. Similar observations were also made in other III–V semiconductor NW arrays grown by SAG,^41,42^ confirming that the NW dimensions are intimately linked to the size of the nucleation site. HR-XRD data further show that the In content in these NWs remains the same for various opening diameters; see Figure S1 in Supporting Information. The unaltered composition was also verified by PL spectroscopy, evidencing that all NW arrays show the same peak energy (see Figure S3 in Supporting Information), irrespective of mask opening size. Figure 1d depicts a high-resolution transmission electron microscopy (HR-TEM) image and corresponding selective area diffraction (SAD) pattern of a typical NW; see Figure 1e, as recorded in a FEI Titan Themis operating at 300 kV. The NW shows a very high density of ultrashort zincblende (ZB) rotational twin domains, as well as stacking defects and even a tendency for random layer stacking, which is typical for SAG-grown GaAs and Ga-rich InGaAs NWs.^38,41,42^ Further below, we elaborate more on the details of the microstructure in relation to the NW diameter and its correlation with hot carrier properties.

**Figure 2:**
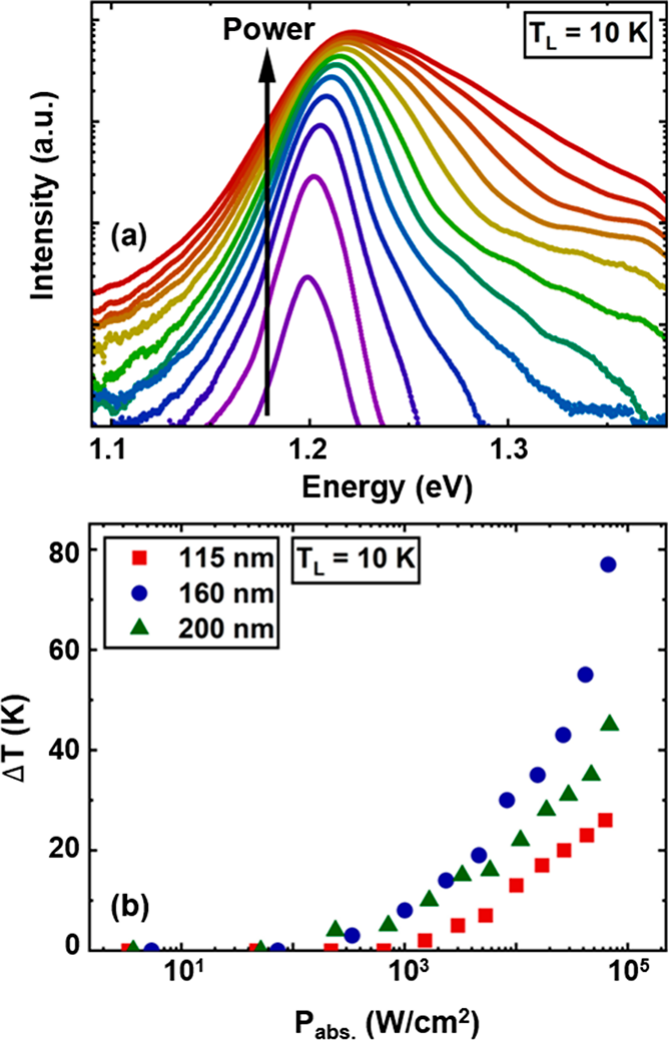
(a) Excitation-power-dependent PL spectra emitted by the InGaAs NWs with a diameter of 160 nm at 10 K. (b) Δ*T* versus absorbed power density at 10 K for NWs with various diameters.

The effects of hot carriers in the passivated InGaAs NWs are investigated by using excitation-power-dependent PL spectroscopy. In these experiments, a 780 nm Ti:sapphire laser with a 2 μm spot size is used to excite hot carriers in the as-grown NW-arrays placed in a He-flow cryostat (10–300 K) and the luminescence emitted from each field is detected by a Princeton Instruments InGaAs CCD. Figure 2a shows typical PL spectra emitted from the InGaAs NWs with a diameter of 160 nm at 10 K under different excitation powers ((10–1.5) × 10^5^ W/cm^2^). To determine the thermodynamic properties of hot carriers in these NWs, the spectra are analyzed using the generalized Planck’s radiation law, which is described by^43,44^
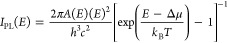
1where *I*_PL_ is the emitted PL intensity, *A* the absorptivity, *h* the Planck constant, *k*_B_ the Boltzmann constant, and *c* the speed of light. The thermodynamic properties of the hot carriers, such as temperature and quasi-Fermi level splitting, are represented by *T* and Δμ, respectively. Figure 2a shows that by increasing the excitation power, the slope of the PL spectra on the high-energy side of the peak position reduces. According to the generalized Planck’s law, since the exponent of eq 1 is inversely proportional to the carrier temperature, the shallower slope of the PL spectrum at higher excitation powers indicates an increase in the carrier temperature.

To determine the average temperature of hot carriers at each excitation power, the PL spectra are fitted with the generalized Planck’s law using the absorption method, which takes into account the change in the sample’s absorption due to band-filling and quantum confinement effects as well as the parasitic absorption induced by the optical setup. The details of this analysis method are reported elsewhere.^45^ Note also that the data are referred to as the real power density absorbed by the NW upon excitation, to allow a direct quantitative comparison of carrier temperature among different samples. This is important because the level of photoabsorption by the NWs varies depending on their dimensions and the array spacing, inducing resonant light-trapping effects, which can result in even greater photoabsorption than in 2D planar structures.^15,16^ Hence, both finite difference time domain (FDTD) simulations (using Lumerical software) and reflectance measurements are performed to account for differences in absorbance of the NW array under different NW diameters (Supporting Information Figures S4 and S5).

The resulting change in carrier temperature with increasing absorbed power density, characterized by the temperature difference Δ*T* (the difference between the temperature of the hot carriers and the lattice), is presented in Figure 2b for the same NWs (blue data) along with data obtained from two other NW arrays with different diameter. All data show consistently the expected increase in the hot carrier temperature at higher excitation power density. It is also evident that Δ*T* varies for different NW diameters, showing that the carrier temperature in thinner NWs (115 nm) is lower than in thicker NWs (160 and 200 nm).

**Figure 3:**
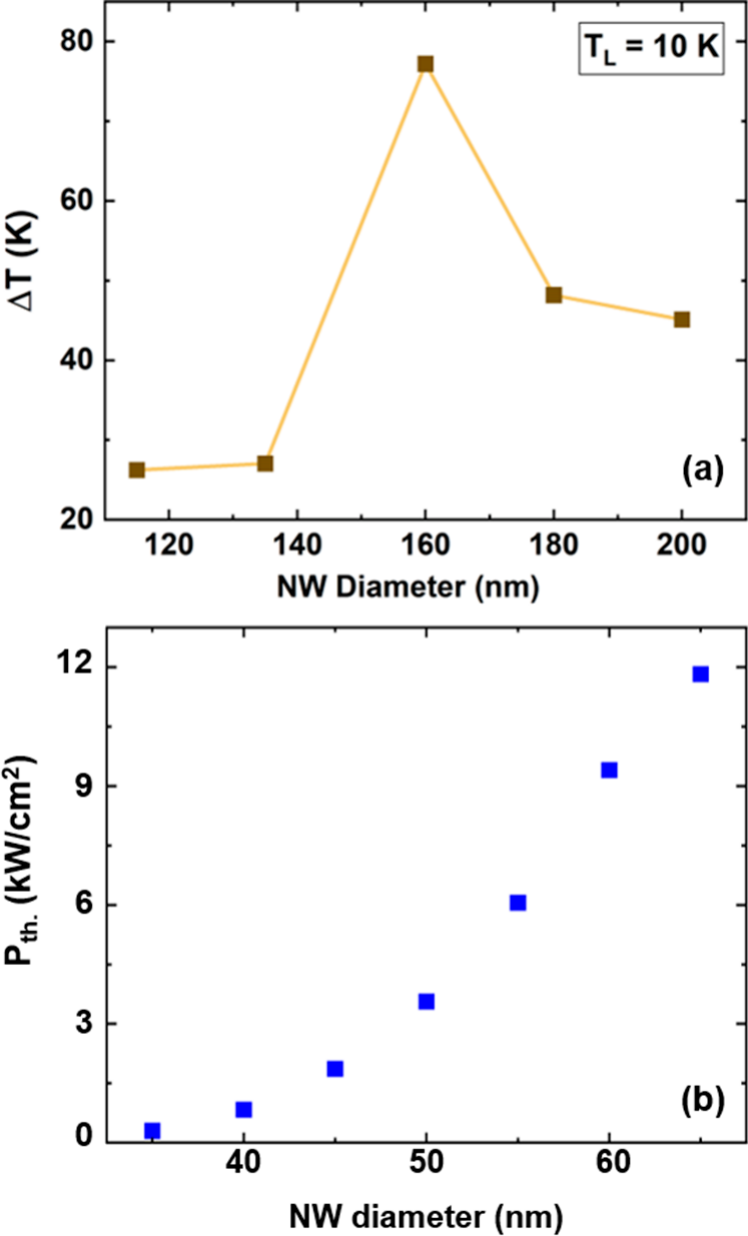
(a) Dependence of Δ*T* on the NW diameter at a given absorbed power density (65 kW/cm^2^). (b) Thermalized power density of hot carriers in the InGaAs NWs as a function of diameter.

To more appropriately compare the temperature of hot carriers at a given absorbed power density, Figure 3a shows the dependence of Δ*T* on the diameter of the NWs at an absorbed power density of 65 kW/cm^2^. The plot indicates that by reducing the diameter of InGaAs NWs from 200 to 160 nm, the hot carrier effects of photogenerated carriers increase. However, by further reducing the diameter of the NWs, the temperature of the hot carriers decreases. Similar results are also obtained under continuous wave excitation (532 nm), as shown in the Supporting Information Figure S6, verifying the same nonmonotonic behavior.

To investigate the origin of this rather intriguing behavior, further theoretical and experimental analyses are carried out to reveal the impact of different electron–phonon scattering rates and carrier dynamics under steady-state conditions in 1D InGaAs/InAlAs core–shell NWs. Hereby, the thermalization power density of hot carriers is determined based on their scattering with LO phonons through Fröhlich interactions and their relaxation via inter- and intra-sub-band transitions in idealized, phase-pure ZB InGaAs NWs.^46^ In this approach, the range of diameters is chosen to be smaller than the actual values of the studied NWs due to computational time. However, all of the diameters are chosen to be larger than the Bohr radius of InGaAs, to simulate the bulk properties of the NWs while taking into account the confinement effect by employing a quantized wave function in the transverse (cross-sectional) plane perpendicular to the axis of the NWs. Figure 3b shows the amount of thermalized power of hot carriers in the computed phase-pure, defect-free InGaAs/InAlAs NWs. We clearly observe that as the NW diameter increases, the amount of thermalized power increases, indicating that weaker hot carrier effects are expected at larger diameters. This behavior mimics, in principle, very well the anticipated trends but cannot account for the decreased Δ*T* observed in the experimental data at the low end of the NW diameter range. Below we show that this discrepancy is directly related to the microstructure since the modeling cannot take the actual defect structure into account.

**Figure 4:**
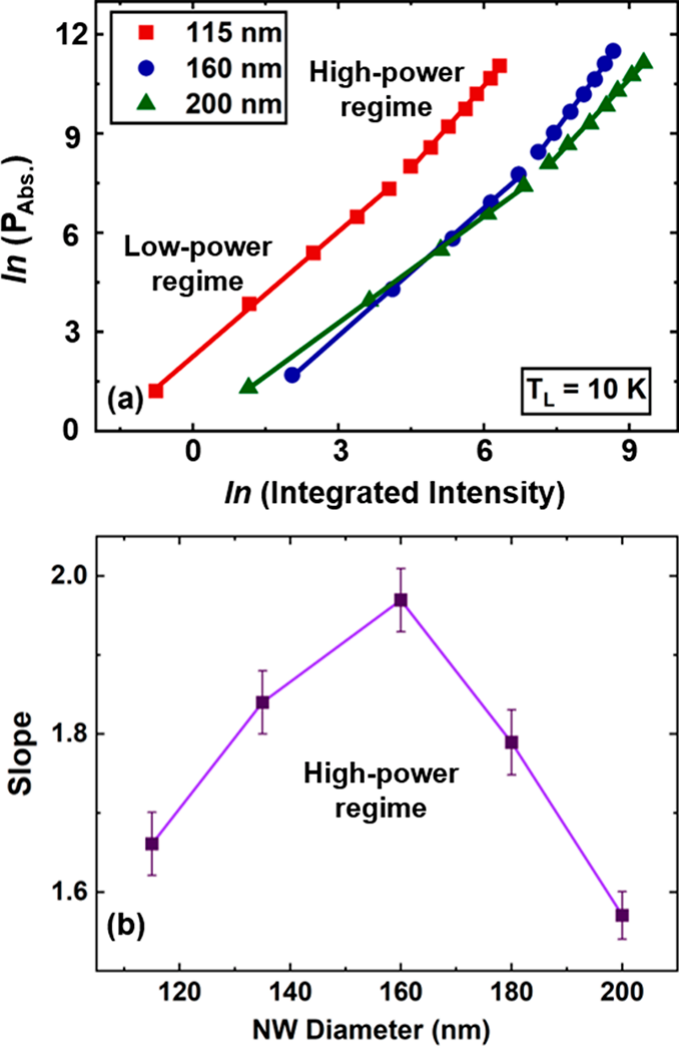
(a) Natural logarithm of the absorbed power density versus the natural logarithm of the integrated PL intensity at 10 K for the InGaAs NWs with various diameters. (b) Slope of the results shown in panel a in the high-power regime, determined by the rate equation analysis.

Another mechanism that can contribute to the formation of hot carrier populations is Auger heating.^45,47,48^ Through this process, the excess kinetic energy of an electron (or hole) is transferred to another particle, promoting it to higher energy states. Although this nonradiative interaction reduces the number of free carriers in the semiconductor, it can benefit hot carrier solar cells by creating hot populations.^48^ The recombination mechanism, and with that the strength of the Auger process, can be studied using the rate equation, which is^49^
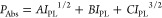
2where the first term is due to Shockley–Read–Hall (SRH), the second term due to radiative, and the third term due to Auger recombination. *A*, *B*, and *C* are the coefficients attributed to these recombination mechanisms, respectively. The relative contributions of these processes can be directly inferred from the scaling characteristics (slope) of the integrated PL intensity vs excitation power.^49^Figure 4a shows the natural logarithm of the absorbed power density as a function of the natural logarithm of the integrated PL intensity, as obtained from three different NW diameters at 10 K. The data show that there are two different slopes for each sample, one at lower excitation powers and one at higher excitation powers. Since robust hot carrier effects on the NW diameter are observed at high excitation powers, we focus on analyzing the mechanism of carrier recombination in this regime.

**Figure 5:**
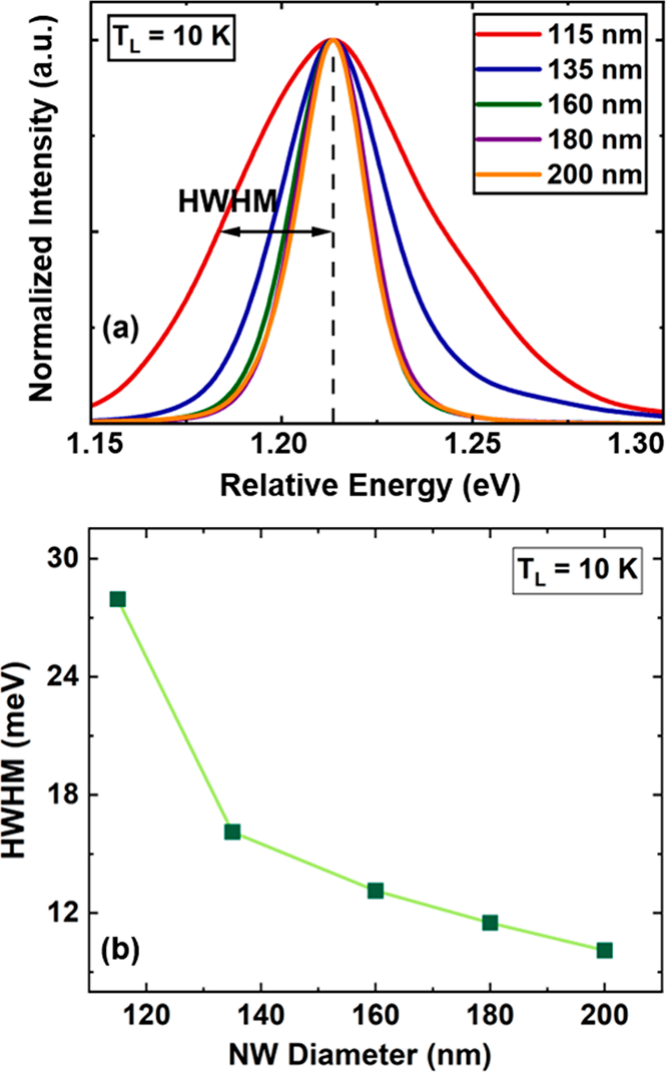
(a) Comparison of the HWHM of the PL spectra for the NWs with various diameters at 10 K. The PL spectra are all shifted to the same peak position on the energy axis shift for better comparison. (b) Results of the HWHM of the PL spectra emitted at low excitation power densities.

**Figure 6:**
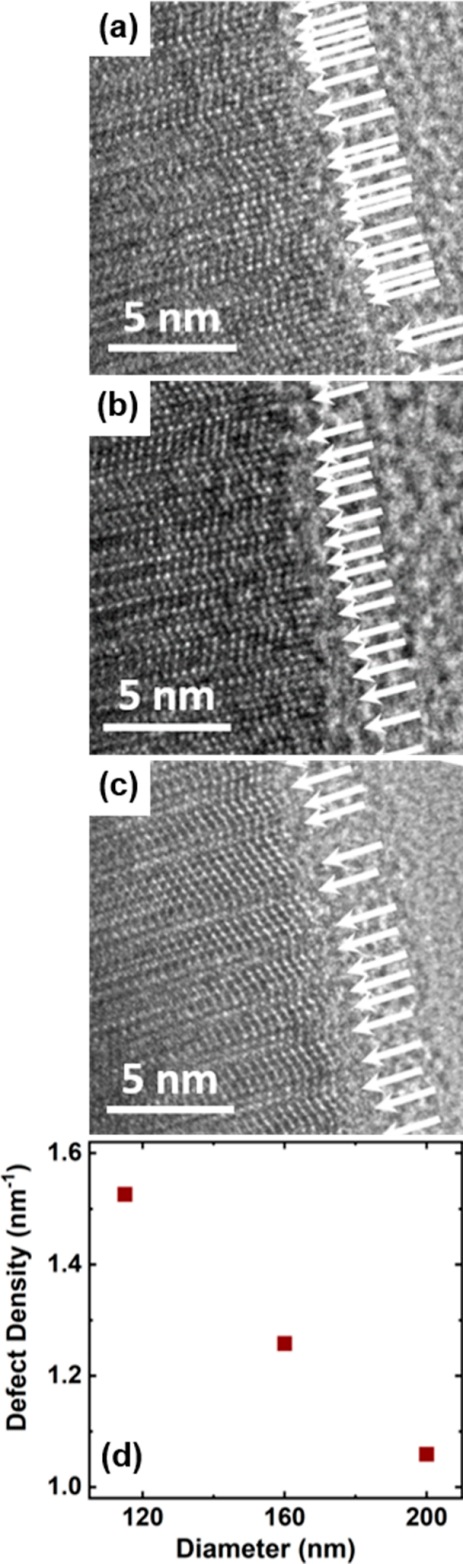
High-resolution TEM images of three different NWs with diameters of 115 nm (a), 160 nm (b), and 200 nm (c). The white arrows indicate stacking defects (twin boundaries) along the growth axis. The defect (twin) density is plotted in panel d versus the NW diameter.

Figure 4b shows the results of the slopes versus NW diameter in the high-excitation power region obtained from panel a, where larger slope indicates more dominant Auger processes.^45,49^ It is seen that by reducing the diameter of the InGaAs NWs from 200 to 160 nm, Auger recombination is obviously enhanced due to stronger charge localization within the NWs. However, with a further reduction in the size of NWs, the effects of Auger recombination decrease markedly. We tentatively attribute this observation to an increased contribution from SRH-type recombination, which lowers the slope and which may originate from the much higher defect densities observed in thinner NWs (see Figures 5 and 6). Therefore, the combination of a higher degree of charge localization, as shown in the modeled thermalized power density, and the stronger Auger-related mechanisms explains the origin of stronger hot carrier effects in NWs approaching diameters of ∼160 nm, where a maximum hot carrier temperature is observed. Something to note in Figure 4b is that slope values indicative of a dominant Auger recombination mechanism are slightly shifted to higher values; i.e., the maximum value determined by the rate equation analysis for the InGaAs NWs is around 2. The origin of this higher value for the Auger-related mechanism in the NWs is currently unclear and requires more advanced, e.g., time-resolved, experiments to better understand the nature of the Auger process at play.

To investigate the origin of weaker hot carrier effects in NWs with diameters of <160 nm, where a reduced Auger recombination at the expense of increased defect-related SRH recombination is anticipated, we place in the following our emphasis on an analysis of defect-related emission and correlated microstructural properties. In particular, the presence of crystallographic disorder in semiconductors can also be seen by sub-bandgap transitions, which are visible as the Urbach tail in the PL spectrum emitted by the material. Figure 5a shows the PL spectra of NWs with various diameters at a low excitation power density. Here, the energy axis is labeled as “relative energy”, where all spectra are slightly shifted in energy depending on diameter and normalized to the same peak intensity for ease of comparison. The PL spectra show that by decreasing the diameter of the NWs, their line width broadening rapidly increases, indicating a deterioration in the crystal quality of the NWs relative to the diameter.^50,51^Figure 6b shows the results of the half-width at half-maximum (HWHM) of the low-energy side of the PL spectra, which therefore excludes any contribution of line width broadening due to hot carrier effects. It is seen that by decreasing the size of the NWs, the HWHM of their emission spectrum increases from ∼8 to ∼28 meV, i.e., a more than 3-fold increase in the size of the Urbach tail.

There are several possible causes for such increased defect densities in the limits of thinner NWs. While surface state defects can be ruled out due to epitaxial InAlAs passivation, point defects or extended structural defects are the most likely sources. Indeed, one can anticipate that thinner NWs grown by vapor–solid SAG processes contain larger defect densities due to enhanced rotational twin formation probabilities, as opposed to thicker NWs, which was observed in recent reports.^52,53^ To demonstrate if this scenario holds also for the present InGaAs NWs, Figure 6a–c depicts HR-TEM images of three NWs with diameters of 115, 160, and 200 nm, respectively. Qualitatively, the images illustrate the same microstructure as observed in Figure 1c, namely, a structure composed of rotational twin ZB domains and many stacking defects. By determining the density of stacking defects along representative sections along the growth axis, it is seen that as the diameter of the NWs decreases from 200 to 115 nm, the density of defects increases monotonically, as shown in Figure 6d. The origin of this dependence on the diameter of the NWs is attributed to the minimization of the Gibbs free energy for the upper facet of the NWs during growth.^54^ As the diameter decreases, the probability of the formation of an upper facet with a diameter close to the critical diameter for twin formation increases, leading to the more rapid occurrence of twins that reduces the surface energy of the facet. This effect also increases the axial growth rate of NWs, leading to the formation of longer NWs with thinner diameters. This is also directly reflected in Figure 1b, which indicates a reciprocal relationship between the NW diameter and their length.

## Conclusion

In summary, we investigated the effects of spatial confinement on the hot carrier properties in core–shell InGaAs/InAlAs NWs using steady-state PL spectroscopy. NWs were grown by selective area growth with diameters controlled between ∼100 and 200 nm, under otherwise fixed alloy composition. The thermodynamic properties of the photogenerated hot carriers in these nanostructures were determined by fitting their PL spectra to the generalized Planck’s radiation law. Experimental results indicated that there is a nonmonotonic behavior of hot carrier temperature versus NW diameter: when the NW diameter decreased from 200 to 160 nm, the hot carrier temperature increased, but for thinner NWs (<160 nm) the temperature of hot carriers decreased. The origin of this nonmonotonic behavior was studied comprehensively by modeling of the thermalization rates of hot carriers in the NWs, analysis of the nature of the recombination mechanism, and the correlated effects of microstructural properties of the NWs. The increase in hot carrier temperature from 200 nm down to 160 nm was, thus, attributed to the combination of slower phonon emission rates and an increase in the Auger recombination rates. However, for very thin NWs (<160 nm), the effects of hot carriers decreased with increasing defect density. This effect leads to higher thermalization rates of hot carriers, thereby reducing the temperature of hot carriers. Our study offers guidelines to the design of nanowire-based hot carrier solar cells to improve hot carrier effects and ultimately to enhance the performance and efficiency of such photovoltaic devices.

## Supporting Information Available

XRD and EDX results of the core–shell NWs, analysis of peak energy position in PL experiments, FDTD simulations, reflection spectroscopy, and hot carrier spectroscopy under continuous-wave excitation (PDF)
