# Supplementary material for: Strong Dimensional and Structural Dependencies of Hot Carrier Effects in InGaAs Nanowires: Implications for Photovoltaic Solar Cells
Source: ACS Appl Nano Mater. 2024 Jan 23;7(3):2817–24. doi: 10.1021/acsanm.3c05041 (PMC10863615; doi:10.1021/acsanm.3c05041)
Supplement: Supplementary file 1 — an3c05041_si_002.pdf [file an3c05041_si_002.pdf]

Supporting Information for:

Strong Dimensional and Structural Dependencies of Hot Carrier Effects in InGaAs Nanowires: Implications for Photovoltaic Solar Cells

*Hamidreza Esmaielpour[a, *], Nabi Isaev[a], Imam Makhfudz[b], Markus Döblinger[c], Jonathan J. Finley[a], Gregor Koblmüller[a, *]*

[a] Walter Schottky Institut, TUM School of Natural Sciences, Technical University of Munich, 85748 Garching, Germany.

[b] IM2NP, UMR CNRS 7334, Aix-Marseille Université, Marseille 13013, France.

[c] Department of Chemistry, Ludwig-Maximilians-University Munich, Munich, 81377, Germany.

* Corresponding authors: *hamidreza.esmaielpour@wsi.tum.de*; *gregor.koblmueller@wsi.tum.de*

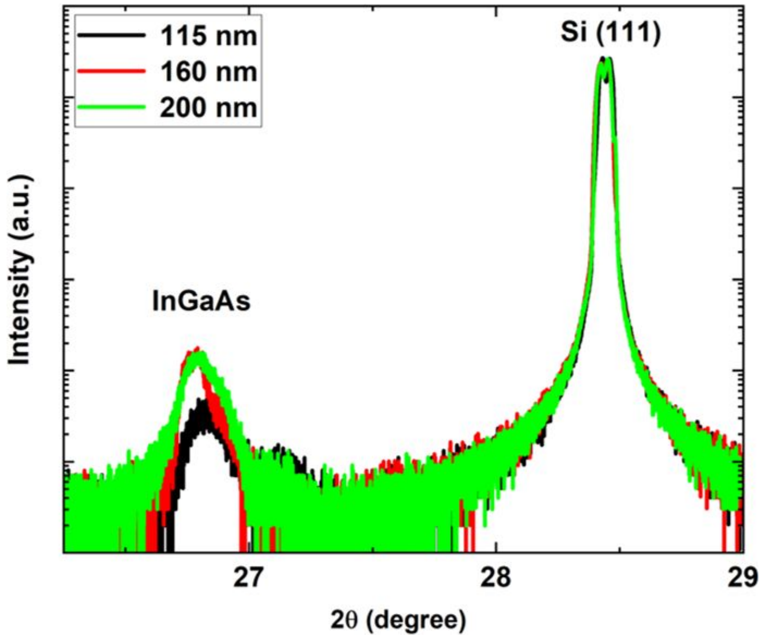

Figure S1. High-resolution X-ray diffraction (HR-XRD) 2theta-omega scans of InGaAs NW arrays with various diameters. The XRD results indicate that the chemical composition of the InGaAs core is the same for various NW diameters.

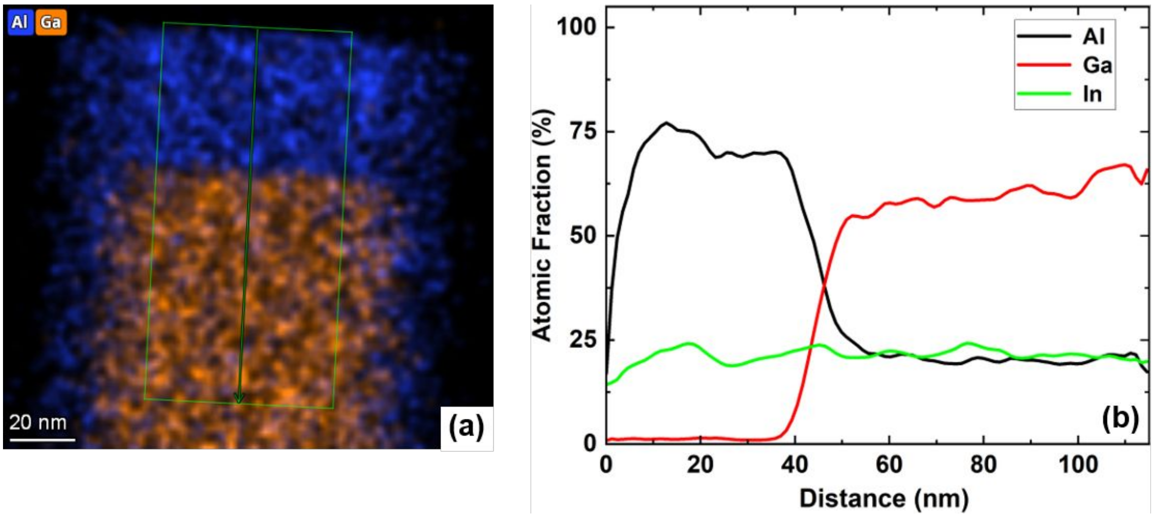

Figure S2. (a) Energy dispersive X-ray (EDX) map of the InGaAs/InAlAs NWs. The region with Al and Ga are shown by blue and yellow, respectively. (b) Atomic fraction of various elements in the NW versus the distance from its tip. According to the atomic fraction, the heterostructure is lattice matched across the interface.

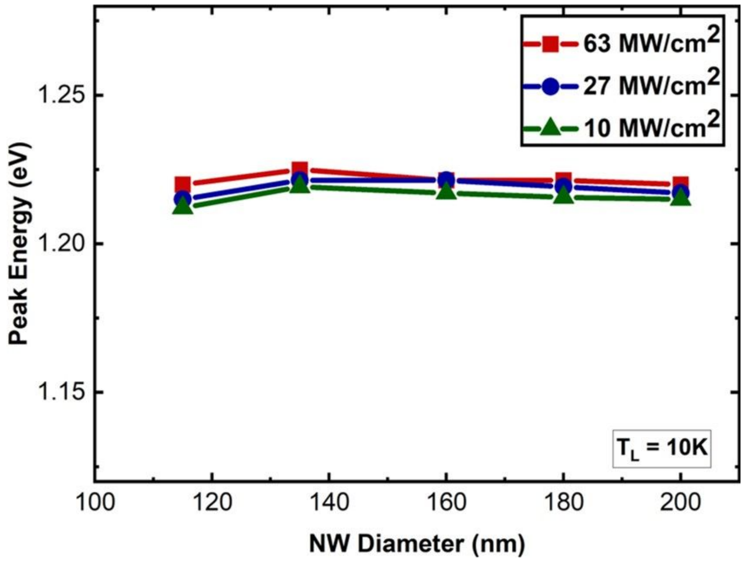

Figure S3. Photoluminescence peak energy position for the InGaAs NWs as a function of their diameters emitted at 10 K under various excitation power densities. The results indicate that the peak energy of the NWs remains the same for various diameters, confirming that the chemical composition of the ternary alloys in these NWs is similar.

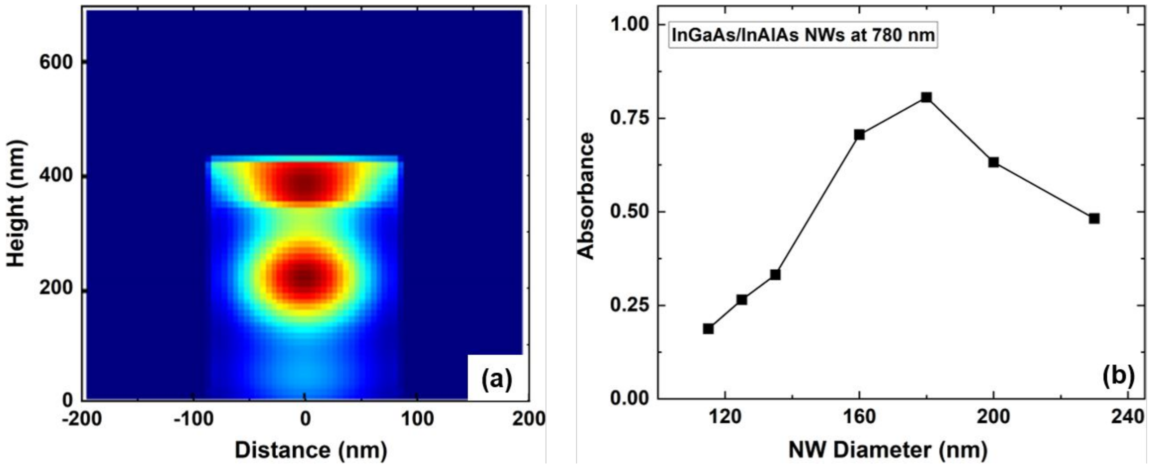

Figure S4. (a) Absorption of the core-shell InGaAs-InAlAs NWs with 200 nm diameter at 780 nm (the wavelength of laser excitation). (b) Total absorption of the NWs versus their diameter. The results are determined by finite difference time domain (FDTD) method and the refractive indices of the ternary alloys are calculated by a method called parametric semiconductor model.[1] The simulation is performed considering a Gaussian beam profile with the same excitation spot size (2 μm) focused on the NW arrays whose dimensions are similar to those of the InGaAs-InAlAs NWs. The pitch size (distance between NWs in the hexagonal array) is 500 nm and is the same for arrays with different diameters. The absorbed power per unit volume is calculated by determining the divergence of the Poynting vector considering the electric field incident on the NWs and the imaginary part of the permittivity of these nanostructures. This analysis is carried out by the Lumerical® program using an analysis tool, called Power Absorbed, which determines the absorbed power by integrating the loss function over the entire simulation volume. In addition, a 3D monitor is set to determine only the photo-absorption by the NWs and exclude the contribution of light absorption by the silicon substrate. To improve the accuracy of the FDTD simulations, small mesh sizes (5 nm along the “X” and “Y” directions and 10 nm along the “Z” direction) are considered to make sharp edges for the hexagonally shaped NWs. The results indicate that there is a maximum of photoabsorption in the NWs at around 180 nm diameter.

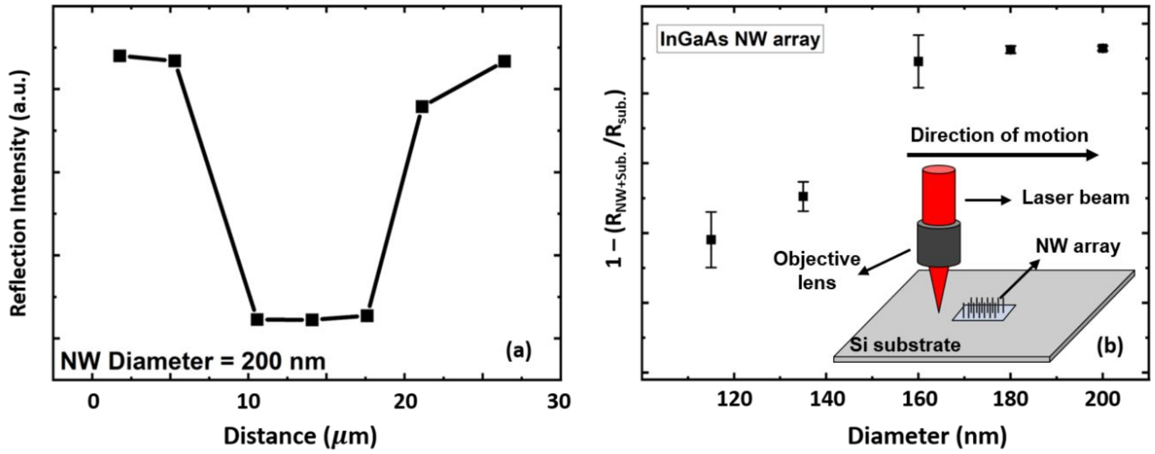

Figure S5. Results of reflectance from the surface of the InGaAs NW arrays using 780 nm concentrated laser for estimating the amount of photo-absorption by the NWs. (a) Reflection intensity versus distance on the sample. The maximum and minimum values are assigned to positions where the laser beam is concentrated on the substrate surface and directly on the NW arrays, respectively. (b) The ratio of the reflection intensities of the NW arrays and the substrate subtracted by 1. The scheme of the experimental setup to measure the reflectance of the sample is shown in the inset of this figure.

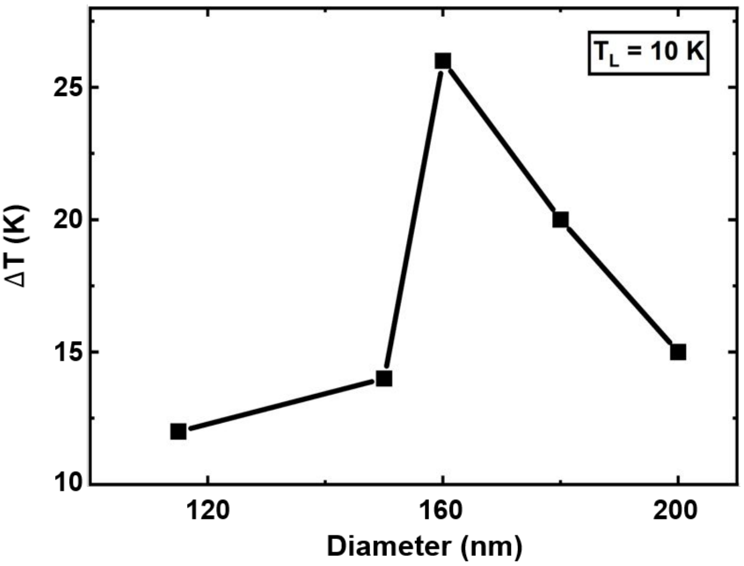

Figure S6. ΔT of hot carriers under a continuous-wave (532 nm) excitation at 10 K versus the NW diameter. The results indicate a similar non-monotonic behavior of the hot carrier temperature observed under the 780 nm pulsed laser excitation.

Reference

[1] Kim, T.J.; Ghong, T.H.; Kim, Y. D.; Kim, S. J.; Aspnes, D. E.; Mori, T.; Yao, T.; Koo, B. H. Dielectric functions of $In_xGa_{1-x}As$ alloys. *Physical Review B.* 2003, 68(11), 115323.
